# Supplementary material for: Unveiling parents’ lived experience with preterm infant care and support in neonatal care units of public hospitals in Nepal: A phenomenological inquiry
Source: PLoS One. 2025 Feb 19;20(2):e0319013. doi: 10.1371/journal.pone.0319013 (PMC12072633; doi:10.1371/journal.pone.0319013)
Supplement: Attachment — Submitted filename: Rebuttal Letter, unveiling experieince of parents .docx [file pone.0319013.s002.docx]

**Rebuttal Letter**

**PLOS One Journal**

**Unveiling parents’ lived experience with preterm infant care and support in neonatal care units of public hospitals in Nepal: A phenomenological inquiry**

**Response to Editor and Reviewers**

Dear Editor and Reviewers,

Thank you very much for providing expert suggestions to revise this manuscript and clarify further. We have included a detailed description below along with the updated manuscript accordingly. Please let us know if you have any questions or need additional information.

Based on the suggestions provided, this manuscript is

- copy edited by a professional native speaker,
- revised following the PLOS ONE manuscript style and
- extensively reviewed by the authors according to suggestions provided by the reviewers and academic editor.

A completed version of PLOS’ questionnaire on inclusivity in global research is uploaded as supplementary information.

Regarding the data underlying findings, most of the data that supports findings of this study is included in the manuscript itself and remaining data are attached as supplementary files: “Data Analysis Matrix, Care and Support”.

Thank you!

**Reviewer’s Comments and Authors Response**

|  | **Reviewer’s Comments and Suggestions** | **Authors’ Responses** |
| --- | --- | --- |
|  | **Abstract** |  |
|  | Please include the duration of the study. | Thank you for your suggestion.  Duration of data collection is mentioned in method section of abstract (clean version of manuscript page 2, Abstract: Methods) |
|  | **Introduction section** |  |
|  | I suggest that the authors enhance the depth and richness of the introduction section by referencing similar studies. For example, I recommend the following article: <https://journals.sagepub.com/doi/full/10.1177/17455057221104674> | Thank you for the thoughtful comments. The introduction section has been revised comprehensively. The authors also included the suggested reference as well as added some more references (also updated references section). Please refer to page 3-6 of the introduction section (clean version of manuscript). |
|  | **Method section** |  |
|  | Please merge the "Research Instrument" section with "Data Collection." | According to the suggestion, “Research Instrument" section is merged with "Data Collection."  Please see Instrument and Data Collection Methods (Page no. 9-10) |
|  | Lincoln and Guba, in their latest edition, have added the criterion of "authenticity" to the four existing criteria for data rigor. Please include this criterion as well, along with an explanation of how it has been implemented. | We are so grateful for your suggestion!  Explanation about application of Lincoln and Guba, "authenticity" criterion in addition to the four existing criteria for data rigor is described in Trustworthiness (page no. 10 to 11) |
|  | Please explain the maximum variation in sampling. | Thanks, maximum variation in sampling is explained in Page 6-7 and shown in Table 1 and 2 (Page 12, 13) |
|  | **Results section** |  |
|  | Regarding the findings, I am fully aware that the authors have deeply immersed themselves in the data and have a better understanding than anyone else. However, as a reviewer, I would like to offer some suggestions on certain themes and sub-themes from a reader’s perspective to enhance clarity and understanding. Please ensure that the abbreviation "EBM" is written out in full upon its first use. Although it was mentioned in the abbreviations section at the end, it was not expanded in the main text when it first appeared. | Thank you! EBM is first time used in page 19 and kept in expanded form there. |
|  | The theme "Support and Care" seems to need a more fitting concept. After reading its content and sub-themes, I realized that it doesn’t merely depict "support and care" but rather presents a continuum that includes both desirable and undesirable support and care. Since a theme, as an abstract concept, should encompass all sub-themes, it would be better to consider a title that aligns more appropriately with this. | We appreciate your suggestion.  We have revised the theme and made it “Diverse Experience of Care and Support”, refer to page 14 |
|  | I believe the sub-theme "Gradual enhancement of infant-parent attachment" can be considered as an outcome and overlaps with the sub-theme "Promotion of infant-parent attachment". If this is not the case, the authors should clarify. | Yes, we value your suggestion.  The "Gradual enhancement of infant-parent attachment" included parents’ experience regarding adequate and inadequate support received for attachment (Answer to how the support for attachment was).  On the other hand, infant-parent attachment as outcome is parents’ feeling of attachment with involvement in hands on care of their PTIs. Therefore, two separate subthemes were kept. |
|  | **Discussion Section** |  |
|  | I suggest that the authors enhance the depth and richness of the discussion section by referencing similar studies. For example, I recommend the following articles: <https://onlinelibrary.wiley.com/doi/full/10.1155/2021/6697659> <https://onlinelibrary.wiley.com/doi/abs/10.1111/nicc.13110> | Appreciating your suggestion, comprehensive revision has been made in the discussion section as suggested by both authors: page 27-32. The suggested resources were also consulted to revise the discussion section. Reference is also mentioned (cited) in the manuscript (Introduction and discussion). |

**Responses to Comments and Suggestions by Reviewer 2**

|  | **Reviewer’s Comments and Suggestions** | **Authors’ Responses** |
| --- | --- | --- |
|  | **Abstract** |  |
|  | While the themes are comprehensive, their presentation could be made more concise, with a clearer distinction between overlapping subthemes (e.g., communication and emotional support). | We appreciate your suggestion. We agree that communication and emotional support are two different and related concepts. In this study, direct emotional support interventions as well as communication in verbal (words, intonation etc.) and non-verbal form (various behaviour responses) in different contexts are also related with emotional support. Therefore, concepts are merged into one subtheme. |
|  | Consider using a visual framework to summarize the relationships between themes. | Thank you for your suggestion!  Themes and subthemes are presented in schematic diagrams showing the relationship (Please refer page 14, clean version of manuscript) |
|  | Expand on how cultural factors specific to Nepal influence parents’ experiences and expectations in neonatal care units. | Appreciating your idea, some findings are discussed relating to care culture in the discussion section, page 26- 29 |
|  | Compare findings more explicitly with existing literature from similar LMICs to provide a broader context. | Thank you for these insightful comments. Authors have incorporated this suggestion in discussion sections, page 26- 29. |
|  | The sample is predominantly mothers, with only a few fathers included. Address this limitation more explicitly in the discussion and suggest strategies for future studies to achieve gender balance. | Realizing your incredible suggestion!  This matter is described in the limitation at the end of the discussion section, page 29-30 of clean version manuscript. |
|  | Consider exploring the implications of variations in parental education and socioeconomic status on their experiences. | Thank you!  Authors have discussed the implications of these variations (parental education and socioeconomic status) in the discussion section page 26- 29. |
|  | Provide more actionable recommendations for healthcare providers and policymakers. For instance, suggest specific training programs for nurses to enhance communication and emotional support. | Appreciating your suggestion, actionable implications and recommendations for nurses and clinicians, policy makers are mentioned in Implication. pages: 31-32 |
|  | Highlight how peer-to-peer support systems could be formally integrated into neonatal care | Integration of peer-to-peer support systems is explained in implication section, page 31 |
|  | Ensure consistency in terminology (e.g., preterm infants (PTIs) versus preterm babies). | Thank you for your valuable suggestion. When the term PTI comes with mothers in verbatim it is kept as baby and in other parts of the manuscript, authors revised and maintained consistency throughout the manuscript. |
|  | Revise some repetitive phrases (e.g., emotional support and care guidance) to improve readability. | Thank you!  This has been revised across the manuscript. After language editing, manuscript has improved readability significantly. |
|  | Correct minor typographical errors in the text. | Realizing the comment, typographical errors are corrected in the text as far as identified. |
|  |  |  |

**Thank you! Reviewers for your valuable and expert support.**
